# Tandem Detethering: A Novel One-Stage Approach Combining Cervicothoracic Cord Release Followed by Filum Terminale Sectioning

**DOI:** 10.3390/jcm14207169

**Published:** 2025-10-11

**Authors:** Natalie Amaral-Nieves, Emilija Sagaityte, Belinda Shao, Shailen Sampath, Rahul Sastry, Prakash Sampath, Petra M. Klinge, Deus Cielo

**Affiliations:** 1 Department of Neurosurgery, Brown University, 593 Eddy Street, APC 6 Building, Providence, RI 02903, USA; emilija_sagaityte@brown.edu (E.S.); belinda_shao@brown.edu (B.S.); rahul.sastry@gmail.com (R.S.); prakash_sampath@brown.edu (P.S.); petra_klinge@brown.edu (P.M.K.); deus_cielo@brown.edu (D.C.); 2 Vagelos College of Physicians and Surgeons, Columbia University, 630 W 168th Street, New York, NY 10032, USA; sgs2187@cumc.columbia.edu

**Keywords:** tandem detethering, filum terminale, tethered cord syndrome, split cord malformation

## Abstract

**Background/Objectives**: We report a prospective series of five patients with symptomatic cervicothoracic spinal cord tethering from prior surgical interventions for acquired and congenital spinal pathologies. Each patient demonstrated incidental radiographic evidence of a low-lying conus or a fatty/thickened filum terminale (FT), suggesting concomitant symptomatic conus tethering as a potential contributor. Therefore, all underwent single-stage “tandem detethering”, consisting of microsurgical release of the cervicothoracic pathology followed by FT resection. **Methods**: Patients’ charts were reviewed for preoperative presentation, imaging, intraoperative findings, surgical details, FT pathology, and six-month outcomes. Results: Preoperative tethering occurred at sites of prior interventions: (i) thoracic arachnoid cyst decompression after Chiari surgery, (ii) cervical lipomyelomeningocele repair, (iii) thoracic ependymoma resection, (iv) syringosubarachnoid shunt placement, and (v) laminectomies for recurrent syrinx. Lumbar MRI demonstrated a low-lying conus in two patients and a fatty/thickened FT in four patients. Intraoperatively, all patients exhibited an abnormal FT (tight, fat-infiltrated, thickened, or dysplastic). No intraoperative complications or neuromonitoring abnormalities were observed. At six months, all patients demonstrated improvement in motor, sensory, pain, and urinary/bowel symptoms. Complications included two pseudomeningoceles requiring repair and one case of recurrent cauda tethering following FT resection. **Conclusions**: In patients with symptomatic cervicothoracic tethering, a concomitant low-lying conus or pathological FT may contribute to symptomatology by perpetuating biomechanical stress and, if not surgically addressed, may limit neurological recovery. This concept provides a rationale for considering tandem detethering under such circumstances.

## 1. Introduction

A tethered cord is an abnormal fixation of the spinal cord at any level. This fixation causes spinal cord traction and alters spinal cord motion, which may lead to axonal stress and straining and reduction in blood vessel diameters, leading to decreased blood flow and subsequent ischemia, oxidative metabolic dysfunction of neurons, and abnormal spinal evoked potentials [1,2]. These metabolic and ischemic changes are thought to be the cause of the symptomatology of tethered cord syndrome (TCS). In children, tethered cord syndrome may present as regression or arrest of developmental sensory, urodynamic, and motor milestones and scoliosis progression. In adults, tethered cord syndrome mostly presents with a triad of back and lower extremity pain, leg weakness, and bladder and bowel dysfunction [3]. Symptoms usually progress in a gradual fashion until disabling factors lead to clinical presentation.

The aim of this prospective patient cohort is to contribute to the literature on rare presentations of secondary spinal cord tethering after previous laminectomies for congenital or acquired pathologies in the cervicothoracic spine or spinal cord. All patients developed new symptoms decades after the initial surgery. Workup with standard spine MRI revealed a low-lying conus and/or thickened fatty FT, consistent with previously unrecognized spina bifida occulta and initially considered incidental. Considering the clinical presentation indicative of concomitant conus medullaris dysfunction, resection of the FT was undertaken in conjunction with cervicothoracic detethering within a single-stage procedure, thereby addressing the potential contribution of additional lumbar tethering. This descriptive study presents our surgical technique, intraoperative observations, histopathological analysis of the resected filum, and six-month clinician-reported outcomes for what we designate as “Tandem Detethering”.

## 2. Materials and Methods

A retrospective chart review was performed on a prospectively collected patient series that underwent “tandem detethering” at a single academic neurosurgical center by the same senior neurosurgeon to ensure procedural consistency. Data collected included detailed preoperative clinical presentation, relevant neuroimaging findings, operative reports, intraoperative observations, histopathological analysis of resected tissue, and a postoperative course. Follow-up assessment included clinical evaluation and imaging at approximately six months after surgery, with variable observation ranging from 8 months to 2 years based on the occurrence of additional procedures. Clinical data and outcomes were recorded using clinician- and patient-reported assessments, following the framework validated in chronic pain trials [4].

Eligibility for tandem detethering required clinical signs of conus medullaris dysfunction—primarily bladder or bowel changes, or focal motor or sensory deficits not fully explained by proximal tethering—together with upper motor neuron findings, suggestive of additional proximal tethering. Patients were selected when both clinical and radiographic evidence indicated the likelihood of dual tethering sites contributing to the neurological presentation.

The tandem detethering technique consisted of two sequential detethering procedures performed during the same operative session. The first stage addressed proximal spinal cord tethering, most commonly in the cervicothoracic or thoracic region, with or without expansile duraplasty to optimize decompression and restore cerebrospinal fluid (CSF) flow and circulation in an attempt to minimize the future risk of retethering. After completion of this stage, a second laminectomy was performed caudal to the conus medullaris–filum terminale junction for microsurgical resection of the FT. The dural closure at this distal site was performed primarily using a running 5-0 Prolene suture, reinforced with fibrin sealant and supplemented by autologous epidural fat grafting to reduce the risk of retethering.

## 3. Results

### 3.1. Surgical Technique

Patients were positioned prone, and both proximal and distal procedures were performed within a single sterile field. Following established practice in split cord malformation (SCM) associated with conus tethering, the proximal tethering is usually addressed first to avoid undue stress or strain on a “fixated” spinal cord when sectioning the FT. Since the patients had prior thoracic surgeries, existing laminectomies were carefully extended by half to a full lamina above and below the target level to expose healthy dura and safely navigate epidural scar tissue. Ultrasound confirmed adequate exposure of the tethered spinal cord segment, and the dura was opened microscopically. Arachnoid adhesions and, when necessary, dentate ligaments were meticulously and sharply resected with the microscissors to restore spinal cord alignment and to untether the tissues while preserving viable nerve roots. In most cases, expansile Alloderm^®^ duraplasty was performed to optimize CSF flow and to increase the dural sac width to minimize neural tissue retethering. The duraplasty was tented up to the facet joint capsules with 4-0 nurolon sutures and followed by complex plastics wound tissue closure.

Distal lumbar exposure was obtained through either a full laminectomy or a translaminar approach, chosen based on the specific pathology described in the case reports. The surgical strategy focused on resecting the FT below the conus medullaris to minimize residual attachment to the spinal cord. The filum was microsurgically isolated from the cauda equina, coagulated, divided with a microscissor, and extracted from its distal dural attachment, thereby maximizing the removal of the FT internum, as previously described [5,6]. Complete filum resection is justified because the FT receives blood from a single conus-level artery; proximal cauterization devascularizes the distal portion, which may otherwise scar and retether. Gentle traction was applied to the severed FT until a ‘snap’ indicated rupture, permitting full extraction. Dural closure was performed using 5-0 Prolene running suture, and an autologous fat graft harvested from the subcutaneous tissue was placed to reduce the risk of postoperative CSF leakage. Complex soft tissue closure was undertaken to optimize wound healing and minimize related complications.

### 3.2. Case Illustrations

#### 3.2.1. Case #1

**Figure 1:**
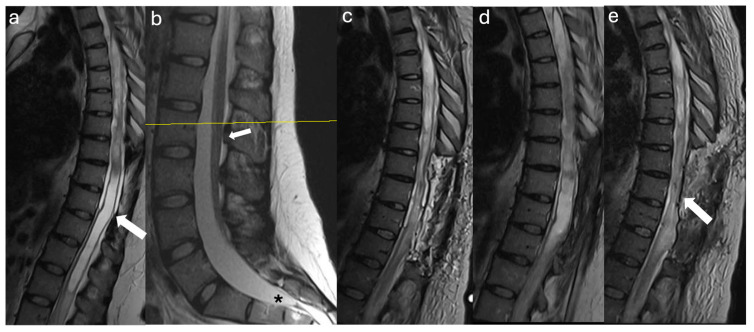
Radiographic Images Case 1: (**a**) Preoperative sagittal thoracolumbar MRI T2 sequence shows a syrinx extending from the thoracic vertebral level T3 to the conus with visible tethering of the spinal cord to the dura dorsally and ventrally at the mid-syrinx level (arrow). (**b**) Preoperative sagittal lumbar MRI T2 sequence shows the conus medullaris (arrow) terminating at mid L2 with a visibly thickened filum terminale at the bottom of the thecal sac (asterisk). (**c**) Postoperative sagittal thoracic MRI T2 sequence before discharge shows laminectomies from T8 to T11 and the expansile duraplasty with marked syrinx reduction at the level and distal to the duraplasty. (**d**) Three-month postoperative sagittal MRI T2 sequence shows syrinx recurrence despite expansile duraplasty with absence of identifiable focal adhesions. (**e**) One-month postsyringopleural shunt placement sagittal MRI T2 sequence shows the shunt at the lower syrinx level (T10 level, arrow) with marked reduction in syrinx size.

**Figure 2:**
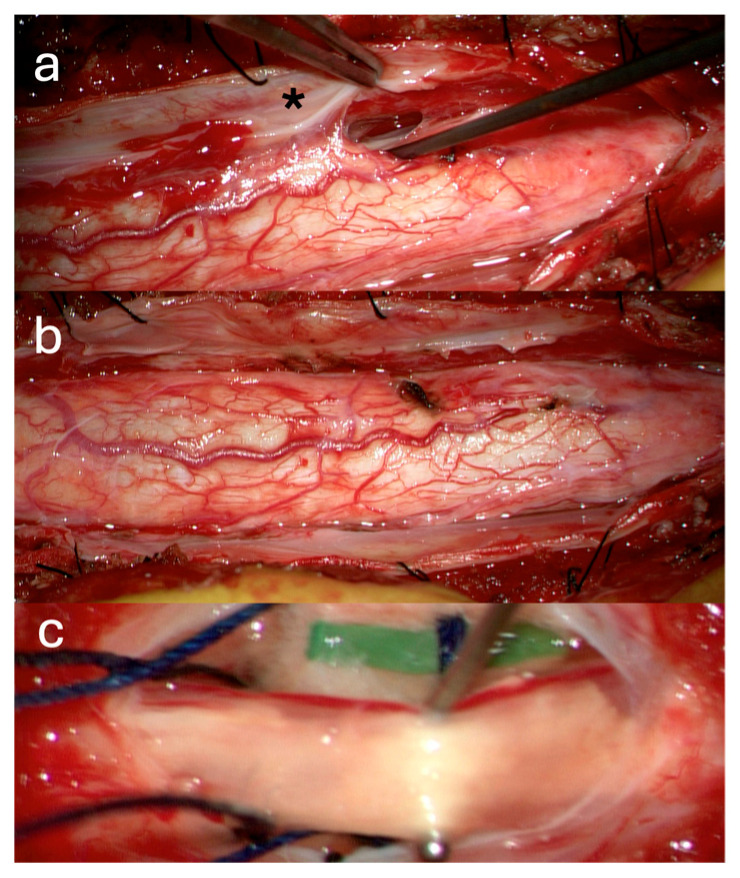
Intraoperative Pictures Case 1: (**a**) Thoracic cord after dural opening (asterisk: arachnoid adhesions). (**b**) Thoracic cord post detethering. (**c**) Exposed Filum.

A 39-year-old female with a history of suboccipital decompression and duraplasty for Chiari malformation, ventriculoperitoneal shunt placement, lumboperitoneal shunt for persistent suboccipital pseudomeningocele, and thoracic laminectomy for arachnoid cyst over a decade ago presented with non-mechanical deep mid and lower back pain of “achy” nature not explained by spinal degenerative changes. The patient was evaluated for progressive left foot drop, bilateral lower extremity numbness and weakness, and urinary retention over several months. Neurological examination revealed 3/5 strength in bilateral knee and foot flexion and extension, 4+ hyperreflexia throughout, and 1-beat foot clonus bilaterally, consistent with myelopathy. Full spine MRI demonstrated a thoracolumbar syrinx with tethering of the spinal cord to the dura via arachnoid adhesions at the level of the syrinx, as well as a low-lying conus at mid-L2 and a thickened FT (Figure 1a,b). The urinary retention, sensation of incomplete emptying, and left foot drop were not fully explained by proximal tethering, suggesting conus dysfunction and lumbar cord tethering. Tandem detethering was performed by first addressing the proximal site via extension of the prior thoracic laminectomy (T8–T11) for arachnoid adhesion lysis and duraplasty with AlloDerm, reinforced with DuraGen and Tisseel, followed by L3 laminectomy for FT sectioning. Intraoperatively, T8–T11 intradural exposure revealed spinal cord tethering to the lateral dura via arachnoid adhesions, consistent with preoperative MRI findings. The spinal cord is visibly tethered and distorted (Figure 2a). Under high magnification, adhesions were sharply divided, restoring CSF flow. The thoracic cord returned to a neutral position with restored vascularity, and expansile duraplasty re-established a patent subarachnoid space (Figure 2b). After the lumbar laminectomy and dural opening, a thickened and calcified FT was isolated from the cauda equina and secured with a nerve hook (Figure 2c). The bright yellow layer on the filum represented subpial microcalcifications, confirmed histologically as fibrovascular tissue with ependymal cells, neuropil containing Rosenthal fibers, and extensive calcifications. In the early postoperative weeks, the patient reported a reduction in back pain, markedly improved spontaneous voiding, stable bilateral lower extremity strength, and a minimally improved foot drop, with MRI showing a reduction in syrinx size (Figure 1c). Three months later, she developed recurrent back pain and progressive motor symptoms along with an MRI that showed syrinx reaccumulation (Figure 1d). Further arachnoid adhesion lysis was deemed inadvisable due to early syrinx recurrence and extensive bilateral adhesions observed intraoperatively, prompting syringopleural shunt placement, which showed radiographic improvement at 1 month (Figure 1e).

#### 3.2.2. Case #2

**Figure 3:**
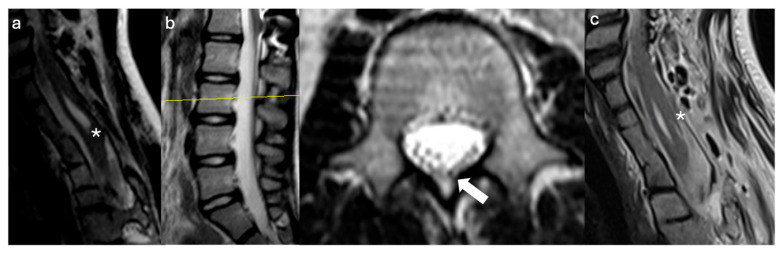
Radiographic Images Case 2: (**a**) Preoperative sagittal cervicothoracic MRI T2 sequence shows a lower cervical cord syrinx with tethering of the cord to the dorsal dura (asterisk). Note the Klippel–Feil deformity of the cervical spine with incomplete segmentation of the lower cervical vertebra. (**b**) Preoperative sagittal and axial lumbar MRI T2 sequences show a visible and prominent filum terminale as indicated by the hypointense T2 spot within the cauda equina (yellow bar, arrow, axial T2). (**c**) Postoperative sagittal cervicothoracic MRI T2 sequence shows decreased syrinx size compared to the preoperative MRI (**a**) and complete detethering of the previously tethered spinal cord segment and established CSF space after expansile duraplasty (asterisk).

**Figure 4:**
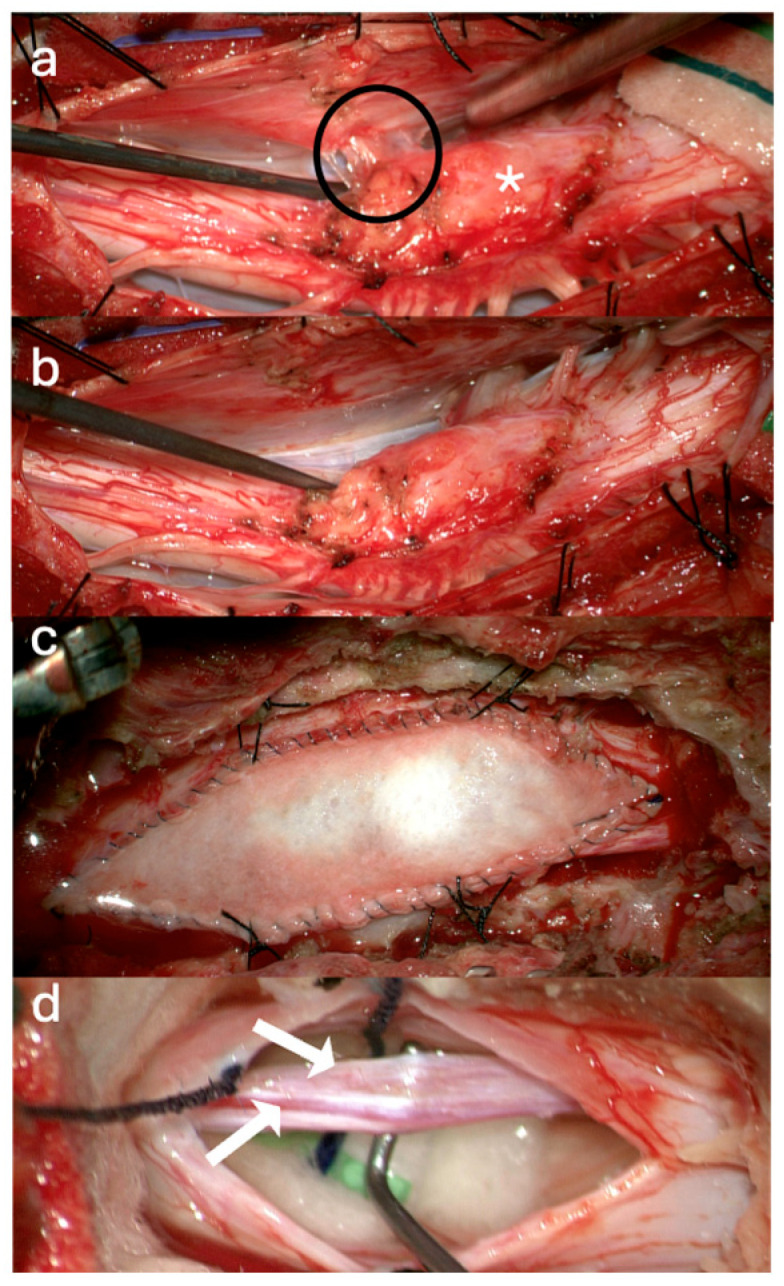
Intraoperative Pictures Case 2: (**a**) Cervicothoracic cord after dural opening (circle: arachnoid scarring with mixed arachnoid and lipomatous tissue, asterisk: syrinx location). (**b**) Cervicothoracic cord post detethering. (**c**) Expansile duraplasty. (**d**) Exposed filum terminale (upper arrow: fat, lower arrow: prominent vein).

A 9-year-old female with Klippel–Feil syndrome and Sprengel deformity, status post cervicothoracic lipomyelomeningocele repair at age one, presented with progressive scoliosis, chronic bilateral lower extremity weakness with easy fatigability, and new-onset bladder incontinence consistent with neurogenic bladder on urodynamic studies. Neurological examination showed intact lower extremity strength with 4+ bilateral hyperreflexia and an extended patellar reflex zone. MRI revealed a C7–T3 syrinx with tethering of the cervicothoracic cord at the prior surgical site due to arachnoid scarring, likely causing the syrinx, and a thickened FT on lumbar axial T2 imaging (Figure 3a,b). Stable cervical syrinx and tethering on prior imaging contrasted with new neurological deficits, suggesting additional distal tethering from a thickened FT and prompting tandem detethering. In a single-stage procedure, prior cervical laminectomies were revised and extended from C3–T3/4, followed by expansile AlloDerm duraplasty to restore the CSF space, then L2–3 laminectomy for FT sectioning. Intraoperatively, arachnoid scarring with mixed arachnoid and lipomatous tissue tethered the cord at the previous lipoma/placode–dural interface (Figure 4a). After adhesion lysis and lipomatous debulking, the cord was released with expansile duraplasty (Figure 4b,c). Translaminar exposure revealed a thickened, fatty, hypervascular FT, isolated from the cauda equina and secured on a nerve hook (Figure 4d). Histopathology of the filum revealed fibrovascular and adipose tissue, confirming congenital filum pathology. Postoperatively, the course was uncomplicated aside from transient headaches and mild incisional staining without CSF leak. At six months, the patient resumed spontaneous voiding and reported mild improvement in leg weakness with extensive physical therapy, though residual back and leg pain persisted, likely related to hypermobile Ehlers–Danlos syndrome and prior scoliosis fusion. The postoperative MRI demonstrated reduced syrinx size and no residual tethering (Figure 3c).

#### 3.2.3. Case #3

**Figure 5:**
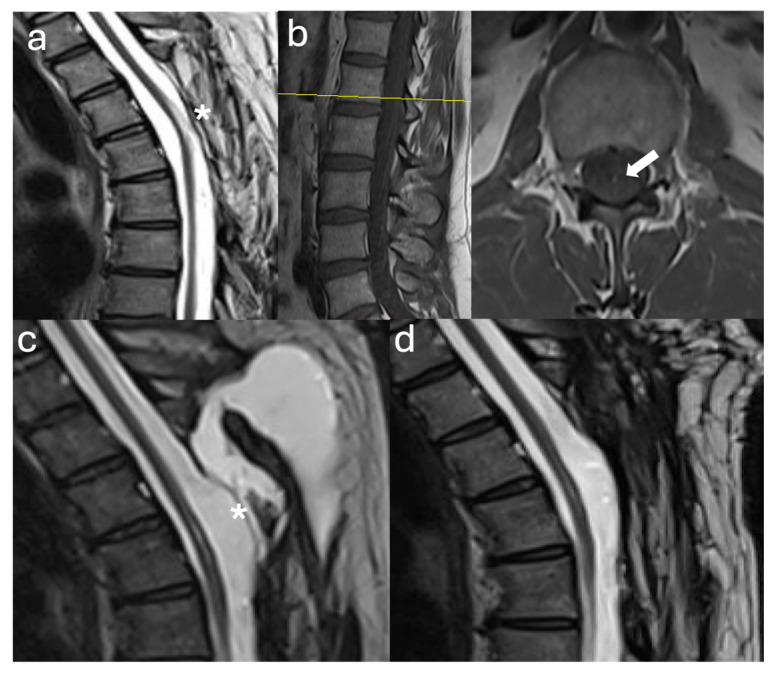
Radiographic Images Case 3: (**a**) Preoperative sagittal thoracic MRI T2 sequence shows fusiform cord atrophy with expansion of the epidural space and tethering of the spinal cord to the dura at the prior tumor resection cavity at the T4–5 level (asterisk). (**b**) Preoperative lumbar MRI T1 sequence shows a prominent and hyperintense appearing filum terminale on sagittal and axial sequences below the conus level that terminates at L1 (yellow line, arrow on axial sequence). This is suspicious for a dysplastic conus/filum transition. (**c**) Postoperative sagittal thoracic MRI T2 sequence shows a patulous cerebrospinal fluid space with complete detethering of the spinal cord after expansile duraplasty (asterisk) and a T3–T6 sizeable midline fluid collection posterior to the duraplasty consistent with a pseudomeningocele. (**d**) Thoracic MRI T2 sequence after the repair of the pseudomeningocele shows complete resolution of the dorsal fluid collection.

**Figure 6:**
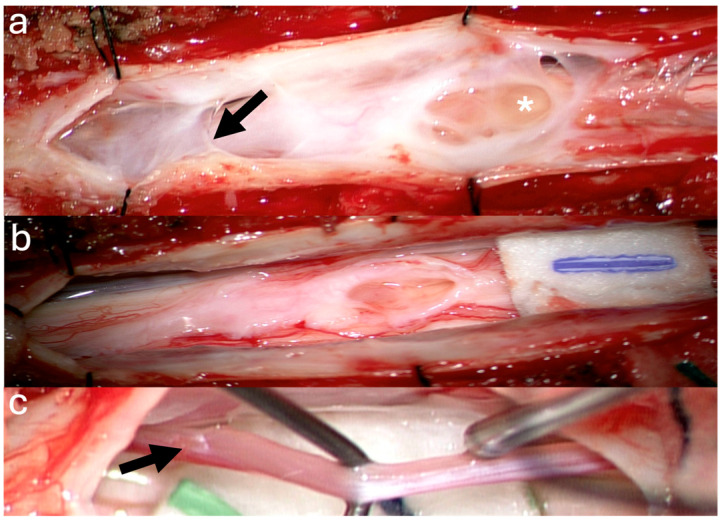
Intraoperative Pictures Case 3: (**a**) Thoracic cord after dural opening (asterisk: extensive bilateral arachnoid adhesions and scarring, arrow: secondary arachnoid cyst). (**b**) Thoracic cord post detethering. (**c**) Exposed filum terminale (arrow: conus).

A 48-year-old male with prior L4–5 endoscopic discectomy (1 year prior) and T4–T6 intramedullary ependymoma resection (20 years prior) presented with fluctuating, non-dermatomal bilateral lower extremity numbness, new lower and mid-back pain, difficulty ambulating due to leg weakness and imbalance, leg tremors and spasms, baseline right foot drop, and urinary retention with incomplete emptying and frequency for at least six months, with recent progression. Examination revealed preserved lower extremity strength (5/5) except for the right foot drop, loss of toe proprioception, 4+ reflexes in all extremities, increased tone in both feet consistent with spasticity, and a few beats of right-sided clonus. The entire spine MRI revealed T4–5 cord atrophy at the site of prior ependymoma resection and spinal cord tethering at the dural closure site, as well as a thickened FT (Figure 5a,b). The urinary dysfunction was not entirely explained by the thoracic cord tethering in the absence of spinal cord signal changes on MRI imaging. Tandem detethering was offered, including revision of prior T4–T7 laminectomies for thoracic cord detethering with expansile AlloDerm duraplasty to minimize retethering risk, followed by L1–2 laminectomy for FT resection. Intraoperative T4–T7 intradural exposure revealed dense bilateral arachnoid adhesions and scarring beneath the prior tumor resection cavity, as well as a small distal arachnoid cyst (Figure 6a). Adhesions tethering the cord to the dura were microsurgically resected to achieve circumferential release, with residual scar adherent to the pial surface. The arachnoid cyst was fenestrated and resected to further detether the cord (Figure 6b). Lumbar exposure revealed a thickened, gliotic FT isolated from the cauda equina and secured on a nerve hook, with a dysplastic conus–filum transition consistent with embryonic disjunction failure (Figure 6c). Histopathology of the FT confirmed fibrovascular tissue with abundant ependymal and rare ganglion cells. Early in the postoperative period, the patient reported subjective improvement in ambulation, balance, bilateral lower extremity strength, back pain, bladder retention and leg tremors and spasms. Three months later, recurrent urinary symptoms and weakness were attributed to a T3–T6 pseudomeningocele even without mass effect on the spinal cord (Figure 5c). Due to intolerable surgical site pain and positional headaches, the pseudomeningocele was surgically revised with primary repair of a lower defect in the AlloDerm duraplasty, resulting in full resolution of the pseudomeningocele and associated symptoms at six months (Figure 5d). At that time, recurrent lower extremity pain, numbness, and leg spasms prompted repeat MRI, revealing cauda equina tethering to a secondary arachnoid cyst and proximal FT remnant. Detethering restored function to prior postoperative levels three months after surgery.

#### 3.2.4. Case #4

**Figure 7:**
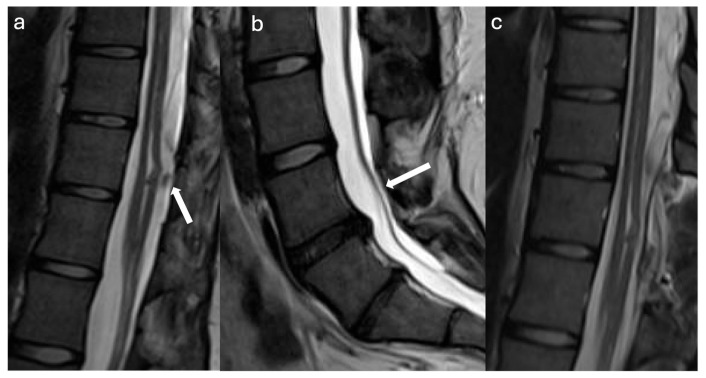
Radiographic Images Case 4: (**a**) Preoperative sagittal thoracic MRI T2 sequence shows a thoracic syrinx with maximal diameter at the T11 vertebral level. The cord shows a focal irregularity at that level most consistent with the syringosubarachnoid shunt exit site (arrow) suspicious for focal arachnoid tethering. (**b**) Preoperative sagittal lumbar MRI T2 sequence shows the previous laminectomy site (arrow) at L4–5 with a dorsal displacement of the cauda equina suspicious of cauda equina tethering most likely associated with a dural retethering of the residual filum terminale at the previous level of filum sectioning (arrow). (**c**) Early postoperative thoracic MRI T2 sequence shows only a slight reduction in syrinx diameter; however, an improvement of the spinal cord dorsal contour after exploration and removal of the subarachnoid shunt catheter is shown.

**Figure 8:**
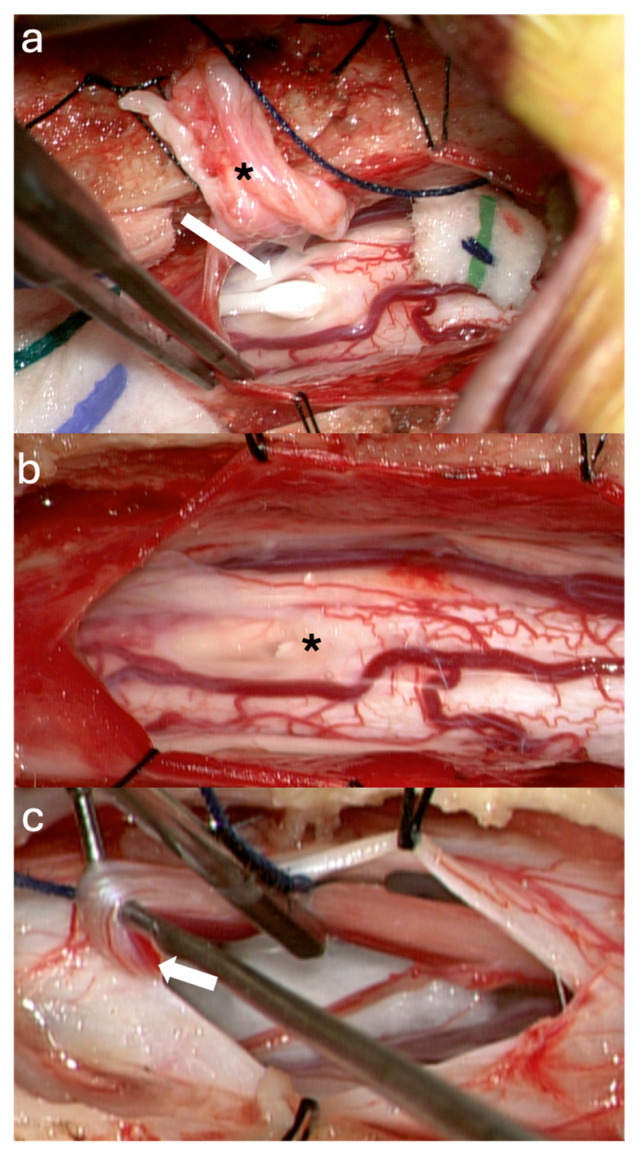
Intraoperative Pictures Case 4: (**a**) Thoracic cord after dural opening (asterisk: extremely thick and dense arachnoid that was resected intradurally, arrow: tethered distal subarachnoid portion of the shunt catheter). (**b**) Thoracic cord post detethering and subarachnoid shunt removal (asterisk: residual intracavitary syrinx shunt portion). (**c**) Exposed filum terminale proximal to the previous level of filum sectioning (arrow: adherence (retethering) of the previously truncated distal filum portion to dura).

A 19-year-old female with prior T11–T12 laminectomy for placement of a syringosubarachnoid shunt and L3–4 laminectomy for FT release in infancy presented with chronic bilateral lower extremity pain, progressive lower extremity weakness and numbness and “achy” low back pain. New-onset urinary hesitancy and retention prompted a urodynamic study, confirming neurogenic bladder. On exam, the lower extremity sensation and motor strength was preserved (5/5). However, the patient had lower extremity hyperreflexia (2+ upper extremity reflexes and 4+ lower extremity reflexes) and spasticity (bilateral foot clonus and increased leg tone). Spine MRI demonstrated possible lower cord tethering at T11–T12, likely due to arachnoid scarring around the distal syrinx catheter, a conus ending at T12–L1, and postsurgical changes at L4–5 with posterior cauda equina displacement, suggestive of tethering from residual FT reattachment (Figure 7a,b). Given radiographic evidence of two separate lumbar tethering sites—at the subarachnoid syrinx shunt and more distally at the residual FT—surgical exploration included T10 laminectomy for detethering at the shunt site, followed by L2–3 laminectomy for residual FT exploration. Revision of the shunt site revealed a dense arachnoid web tethering the cord to the dura and catheter, which was sharply dissected under the microscope, preserving neural structures. Lysis of adhesions and removal of the subarachnoid shunt portion resulted in cord detethering (Figure 8a). The intramedullary syrinx shunt portion was left in situ, allowing it to retract after tubing truncation (Figure 8b). At L2–3, the distal FT was thickened and tightly adherent to the dura at the prior sectioning site, as visualized with gentle elevation using a nerve hook (Figure 8c). Histopathology confirmed dense fibrovascular connective tissue with peripheral nerve and ganglion cells. Postoperatively, the patient gradually improved ambulation over six months with physical therapy, experienced significant reduction in back and leg pain, regained spontaneous voiding, and demonstrated 5/5 lower extremity strength with resolved clonus. Six-month MRI showed slight syrinx reduction and improved cord detethering (Figure 7c).

#### 3.2.5. Case #5

**Figure 9:**
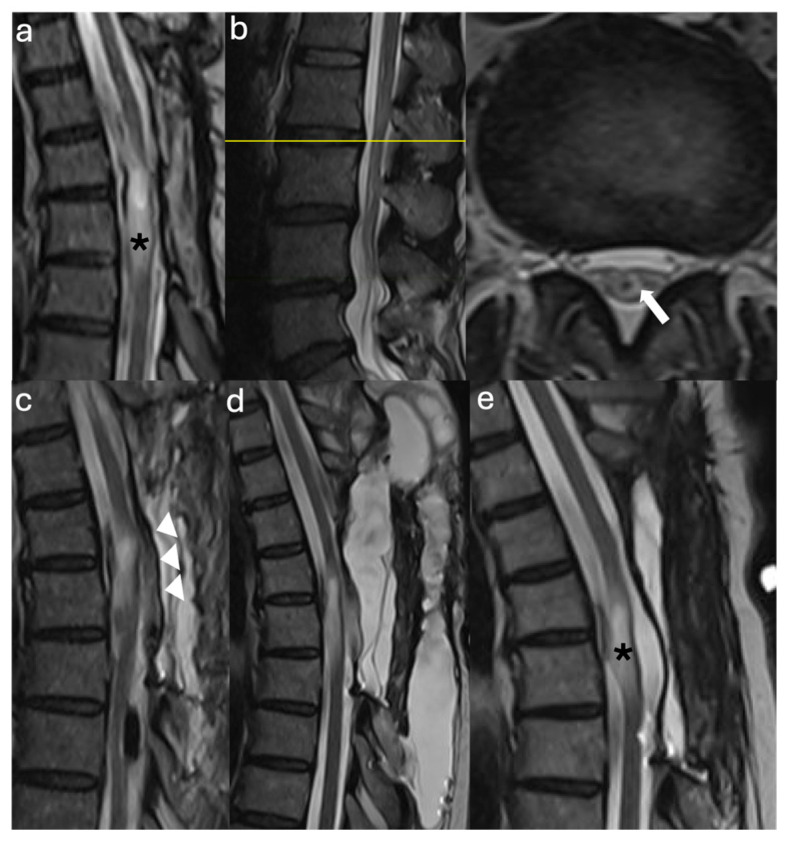
Radiographic Images Case 5: (**a**) Preoperative sagittal thoracic MRI T2 sequence shows syringohydromyelia associated with myelomalacia (asterisk) extending from T4–T6 at the prior T3–T5 laminectomy site for prior syrinx decompression. (**b**) Preoperative sagittal and axial lumbar MRI T2 sequences show a low-lying conus at the mid-L3 vertebral level. The conus tip is seen at the axial sequence at the upper L3 level (yellow bar, arrow). (**c**) Early postoperative sagittal thoracic MRI T2 sequence shows a significantly smaller thoracic syrinx with stable myelomalacia, expansile duraplasty (arrowheads). (**d**) One-month postoperative sagittal thoracic MRI T2 sequence shows a dorsal T3–T6, 4.7 × 3.4 × 11.1 cm sized pseudomeningocele. (**e**) Sagittal thoracic MRI T2 sequence after two duraplasty revisions shows no residual tethering and patulous CSF space posterior to the previously tethered spinal cord along with near resolution of the syrinx with residual pre-existing myelomalacia (asterisk).

**Figure 10:**
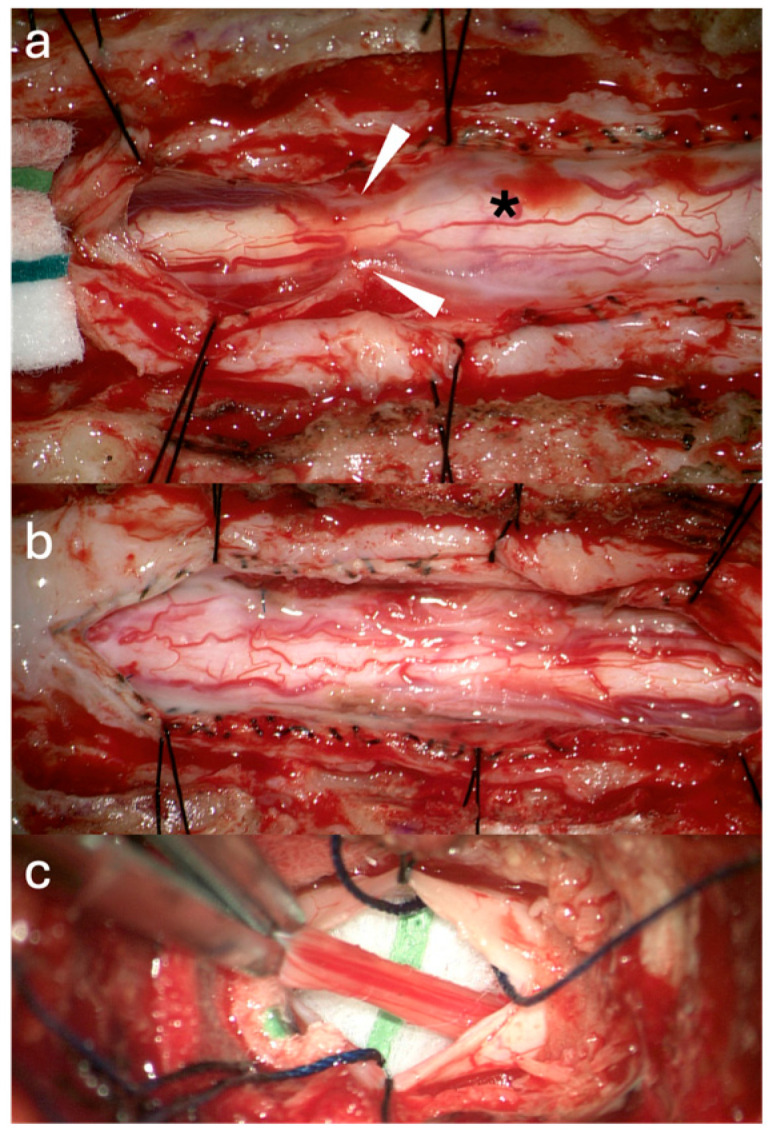
Intraoperative Pictures Case 5: (**a**) Thoracic cord after dural opening (arrowheads: focal T5 arachnoid scar encasing the spinal cord, asterisk: syrinx). (**b**) Thoracic cord post detethering. (**c**) Exposed filum terminale.

A 47-year-old female with prior T3–T5 laminectomy for intradural syrinx decompression one year earlier presented with progressive bilateral lower extremity weakness, loss of ambulation, complete bowel and bladder incontinence, and severe mid-back pain, leaving her bedridden. Examination revealed 3+/5 weakness in the right upper and lower extremities compared to 4/5 strength on the left side, and 2+ reflexes throughout. The entire spine MRI showed T4–T6 syringohydromyelia and suspicion of focal arachnoid adhesions at the lower aspect of the syrinx, a low-lying conus at L3, and a thickened FT (Figure 9a,b). Although the examination was equivocal, the patient’s severe pain and disability made it difficult to determine the relative contribution of proximal versus distal spinal cord tethering. However, the low-lying conus medullaris raised concern for additional conus and lumbar spinal cord tethering contributing to the debilitating and rapidly declining symptoms. Therefore, tandem detethering was offered, which involved revision and extension of the previous T4–T6 laminectomies with expansile Alloderm duraplasty for thoracic spinal cord detethering and restoration of CSF flow at the thoracic level, followed by L2–3 laminectomy approach for FT resection. Thoracic dural exposure revealed a focal T5 arachnoid scar encasing the spinal cord distal to the syrinx, which was microsurgically released and resected (Figure 10a). Post-resection, the thoracic cord visibly expanded following release of the tethering arachnoid adhesions (Figure 10b). L2–L3 laminectomy exposed the FT, which was sectioned below the conus and pulled to release its distal dural attachment. Congested filum veins, fatty degeneration, and a dysplastic conus–filum transition indicative of embryonic disjunction failure were observed and later confirmed histologically as abundant fatty deposition and filum hypervascularity (Figure 10c). The patient experienced slight improvement in bilateral lower extremity strength but remained non-ambulatory due to persistent disabling back pain, with reported improvements in sensation and voiding. She participated in extensive rehabilitation. An early postoperative MRI showed reduction in syrinx size with patulous CSF space (Figure 9c). One month later, she developed severe and intolerable positional headaches, urinary urgency, pain, and incisional fluctuance. The MRI revealed an extensive T3–T6 pseudomeningocele (Figure 9d). Two thoracic duraplasty revisions were performed to repair a focal CSF leak at the mid-lateral duraplasty site, allowing resumption of rehabilitation and reducing the syrinx (Figure 9e). Despite radiographic resolution of tethering, she continued to experience chronic back pain, likely from degenerative spine disease and secondary kyphoscoliosis, and was monitored for potential spinal fusion.

## 4. Discussion

We present a series of patients with cervicothoracic spinal cord tethering to the dura secondary to prior interventions for congenital or acquired spinal cord pathologies, along with the finding of a low-lying conus and/or thickened, fatty FT. Although the latter was initially considered an ‘incidental’ finding on presentation, patients manifested symptoms suggestive of conus or lower lumbar cord involvement that were not fully attributable to the proximal tethering alone. This raised concern that addressing only the cervicothoracic lesion might result in incomplete symptom resolution, thereby challenging the notion that these findings were truly incidental. Suspecting additional biomechanical stress from FT pathology, we chose to address both lesions: the obvious cervicothoracic tethering and the potential conus/lumbar spinal cord tethering associated with the radiographically shown FT abnormalities pointing towards spina bifida occulta. Our series illustrates what we term a “tandem detethering” approach. While this study does not allow us to establish the efficacy or overall safety of the approach, we discuss that no complications were observed beyond the expected spectrum associated with either procedure performed individually.

### 4.1. Pathophysiology and Histopathological Evidence Supporting Filum Terminale Resection

The clinical presentation of the triad of lower extremity and back pain, lower extremity neurological symptoms, and bowel/bladder manifestations establishes the clinical diagnosis of TCS despite minimal radiological criteria. Proposed treatment for TCS involves release or resection of FT via laminectomy [7,8,9]. Efforts to create a pre-operative scale to help predict outcomes in patients with TCS after FT release have shown a 68% symptom improvement 1 year post-surgery and an accurate outcome prediction in 82% of patients [9].

In support of our tandem approach, all patients indeed were found to have intraoperative evidence of filum pathology, i.e., a tight, thickened, fatty, or dysplastic FT. Also, the FT histopathology findings such as hypervascularity, fatty infiltration, and/or abundance of embryonic spinal cord tissue support a congenital nature of the FT pathology due to disjunction failure after retrogressive regression [5,10,11]. Prominent vessels, as seen in Case 5, have been deemed a possible biomarker for TCS in a pediatric series, as this could imply blood pooling from the biodynamic alterations and tethering causing venous hypertension and congestion [10,12]. Calcifications, as observed in Case 1 histopathology, may reflect mechanical overuse of the FT—similar to tendonitis—and have been described in adult-onset tethered cord, though previously overlooked [5,13,14]. To support the biomechanical implications of spinal cord tethering in the setting of a pathological filum, bench tests have shown pathological stress and strain on the spinal cord even when the conus medullaris is positioned at a normal level. This phenomenon is attributed to the loss of the FT’s intrinsic elasticity and “shock-absorbing” capacity, resulting in altered spinal cord motion and increased vulnerability to traction-related injury [12]. These insights provide the biomechanical and pathophysiological rationale for incorporating FT resection into our tandem detethering approach. We speculate that after detethering of an upper spinal cord level, residual traction forces transmitted through an abnormal FT may continue to stress the neural tissue, thereby potentially limiting neurological recovery. By removing the FT during the same operative session, we aim to reduce the likelihood of incomplete symptom resolution in patients with dual tethering pathology [12].

### 4.2. Relationship to Split Cord Malformation and Surgical Approaches

Our proposed tandem detethering approach mirrors the treatment of split cord malformation (SCM), which was present in two of our cases. Kim et al. (2013), Akay et al. (2004), Quinones-Hinojosa et al. (2004), and Elmaci et al. (2001) delineate approaches to SCM involving detethering or transection of adhesions in the upper spine prior to release of the FT [15,16,17,18]. However, Oh et al. and D’Agostino et al. (2021, 2019) took a reportedly unique approach in a patient with tethering above the terminal spinal cord, prompting FT detethering preceding septal removal—an atypical approach compared to usual recommendations [19,20]. Other reported cases of SCM with TCS have involved pathology also seen in our cases, such as low-lying conus, lipomas, Chiari malformation, and scoliosis [21,22]. Tandem detetherings have been reported for double-detethering involving the lumbar spine with kyphosis correction, SCM with lipoma and syrinx in an infant, and tethered cord with two lipomas [23,24,25]. Ramdurg (2017) described tandem lipomas in the lumbosacral and FT regions, both of which were addressed in a single procedure [26]. Other unique presentations in the literature included tethering at proximal but distinct sites due to double MMC [27]. Also, Xu et al. (2020) surgically corrected a double FT in the context of SCM [28].

Although reported in the literature, this remains controversial, especially in adults and in cases of acquired tethered cord following surgeries performed decades earlier, as seen in our mixed cohort. We recommend addressing the upper pathology first before completing conus detethering, following the pediatric SCM approach in which premature FT release can cause upward cord movement and neurological stress at the split cord defect fixation. In addition, Barutcoaglu et al. (2015) found increased hyalinization and loss of elastic fibers in the FTs of 33 SCM patients, like our previously discussed findings in occult TCS [29].

### 4.3. Surgical Risks and Complications

The potential risks and benefits of tandem detethering merit careful consideration in comparison to cervicothoracic detethering alone or isolated filum release. Throughout all cases, we did not encounter any intraoperative neuromonitoring changes suggestive of undue traction or stress on the spinal cord during the combined detethering procedure. Likewise, no intraoperative complications were observed, and patients, with the exception of pain, did not experience any new or worsened neurological deficits in the immediate postoperative period. While we did not encounter intraoperative complications in our series, the addition of a second-level procedure inherently extends operative duration (6 to 9 h; 2 to 3 h) and anesthetic exposure, which may translate into increased perioperative risks. These include heightened challenges in pain control, longer hospital stays, and the downstream risks of prolonged immobility such as thrombosis, deconditioning, and extended rehabilitation requirements. Additionally, the neurological impact of addressing two tethering sites in a single procedure currently remains incompletely understood. Balanced against these risks are the potential benefits of a single-stage procedure: addressing both the cervicothoracic and filum-related tethering simultaneously, reducing the risk of delayed recovery or incomplete improvement from untreated tethering, and minimizing residual biomechanical strain on the spinal cord transmitted through a pathological FT [30,31]. As discussed above, this strategy, conceptually analogous to the management of SCM, where both axes of tethering are corrected to optimize outcomes, may support more complete recovery in selected patients [15,16,17,22,23,24,25,26,27,32]. While the neurological risks and potential benefits of this novel approach require evaluation in larger prospective, controlled case studies, we did not observe any of the aforementioned perioperative complications. Hospital stays averaged 3–8 days, primarily determined by the cervicothoracic procedure, and were comparable to those reported for similarly complex spine interventions, including instrumented fusion cases with prolonged operative times [33].

However, our case series poses a theoretically higher risk of complications due to the complex nature of the cervicothoracic pathologies and the double durotomy sites, i.e., increased risk of CSF leak. Pan et al. (2023) compared perioperative complications in pediatric patients with simple and complex TCS and found that complex TCS, such as intraspinal lipoma, lipomyelomeningocele, scar formation after myelomeningocele repair, dermal sinus tracts, myelocystoceles, SCM, and teratomas have a higher risk of complications, including CSF leak, need for permanent CSF diversion, and need for retethering surgery [34]. Two patients required additional surgery to repair thoracic pseudomeningoceles associated with CSF leaks at the duraplasty site [35]. No CSF-related complications occurred at the lumbar laminectomy site for FT resection. These pseudomeningocele complications cannot be attributed to the lumbar detethering or extended operative time, but rather to prior extensive intradural procedures, where altered dural integrity and significant epidural scarring likely increased the risk of delayed duraplasty dehiscence. None of our patients showed retethering of upper cervicothoracic sites during the observation periods, but 1/5 patients had lumbar retethering within three years. Literature has shown FT retethering risks ranging from 1.7 to 8.2%, which might correlate to postoperative persistent low-lying conus, decreased spinal cord thickness, and unchanged syrinx size [34,36]. A persistent postoperative low-lying conus and recurrence of symptoms in Case 3 prompted a second lumbar detethering intervention.

### 4.4. Limitations

This study has several limitations. First, it is a retrospective chart review with a short-term follow-up period. In chronic and ultimately disabling conditions such as tethered cord syndrome, the primary surgical goal is to halt progression rather than to produce rapid, curative effects. Beyond six months, outcomes may become increasingly confounded by comorbidities related to chronic disability and pain, as well as by social and lifestyle factors or other confounding influences that accumulate over longer intervals that dilute the causal link to the initial intervention. Indeed, several studies suggest that short-term assessments—sometimes as early as three months—may provide the most reliable and robust measure of change attributable directly to surgery. Trial designers frequently view 3- to 6-month as a “safe” window to detect the direct effect of the surgical intervention. Second, we further acknowledge that our study does not employ disease-specific validated outcome scales; however, it is critical to recognize that no such instruments exist for tethered cord syndromes. The fluctuating, multi-limb, and non-dermatomal symptomatology encompassing unique and a variety of sensory–motor anomalies, often asymmetric, characteristic of complex tethered cord patients falls outside the scope of currently available scales, which were designed for more uniform neurological or pain conditions [37].

Furthermore, histopathological images of the FT are not presented here, as they have been published and described in detail previously [12]. Finally, the absence of a control population precludes definitive proof of concept, and our findings should therefore be interpreted as observational rather than conclusive. While we believe our case series supports the proposed rationale—an approach historically recognized in the management of SCM—the results remain both descriptive and speculative in nature. Nonetheless, the discussion provides the clinical and scientific context underlying our decision-making and is intended to contribute relevant evidence to support and advocate for consideration of this approach in selected patients.

## 5. Conclusions

Although our patient cohort does not establish causality or superiority of this tandem approach, we advocate for a thorough evaluation of the conus level and FT on imaging, along with careful clinical assessment for conus-related symptoms in patients with any primary or acquired spinal cord tethering pathologies.

## Data Availability

The original contributions presented in this study are included in the article. Further inquiries can be directed to the corresponding authors.

## Abbreviations

The following abbreviations are used in this manuscript:

FT	Filum terminale
TCS	Tethered Cord Syndrome
MRI	Magnetic Resonance Imaging
CSF	Cerebrospinal Fluid
SCM	Split Cord Malformation
